# Prevalence of posttraumatic stress disorder among adolescents in school and its impact on their well-being: a cross-sectional study

**DOI:** 10.11604/pamj.2021.39.54.27419

**Published:** 2021-05-19

**Authors:** Khalid Astitene, Amina Barkat

**Affiliations:** 1 Health and Nutrition Research Team of the Mother and Child Couple, Faculty of Medicine and Pharmacy of Rabat, Mohammed V University, Rabat, Morocco,; 2 Department of Medicine and Neonatal Resuscitation, Ibn Sina Children's Hospital, Faculty of Medicine and Pharmacy of Rabat, Mohammed V University, Rabat, Morocco

**Keywords:** Adolescence, associated disorders, posttraumatic stress disorder, school students, traumatic events

## Abstract

**Introduction:**

anyone can develop posttraumatic stress disorder (PTSD) following a traumatic event; this disorder can develop comorbid PTSD disorders such as anxiety and depression, which could seriously interfere with the daily life of the adolescent who was to be the subject of our study by evaluating the prevalence of PTSD in public schools and also evaluating the impact of this disorder.

**Methods:**

the survey was carried out during the period from March to June 2017. Participants were selected for a cross-sectional survey. Standardized questionnaires (life events checklist, CPTS-RI, STAIY and CDI) were used. The independent variables were investigated using binary logistic regression analyzes which were performed to investigate factors associated with PTSD.

**Results:**

the number of students was 982 adolescents with an age of 12 to 17 years (14.98 ± 1.49) and the participation rate was 88.69% (n = 871). A high prevalence of PTSD was found with 19.3% (n = 168). The factors independently associated with PTSD included being a girl (adjusted odds ratio (AOR) =2.113, 95% C.I =1.015-4.399, p=0.046), having a middle school level (AOR =5.765, 95% C.I =2.262-14.692, p<0.0001), sleep interrupted (AOR =0.142, 95% C.I =0.027-0.745, p=0.021), guilt (AOR =27.378, 95% C.I =6.835-109.663, p<0.0001), difficulties of memory (AOR =0.157, 95% C.I = 0.071-0.346, p<0.0001), and difficulties of concentration (AOR =0.041, 95% C.I = 0.004-0.392, p=0.006). Among adolescents in school with PTSD, anxiety had 79.1% (n = 133) and depression had 51.1% (n = 86).

**Conclusion:**

the prevalence of PTSD and comorbid anxiety and depression was high among educated students. Factors associated with PTSD included being in college, being a girl, and having guilt. It is necessary to adapt suitable treatments immediately after a traumatic event or during the disease.

## Introduction

Posttraumatic stress disorder (PTSD) has been the subject of several research studies in recent years on the epidemiological, neurobiological or therapeutic levels, but there are few studies in children and adolescents of school age, especially in developing countries. Posttraumatic stress disorder is classified as a severe anxiety disorder and is a major public health problem around the world. When a person has been exposed to an assault or a sudden threat involving his life or his physical or mental integrity, he immediately presents an alarm reaction to face this assault [1]. According to the DSM-IV classification [2], the diagnosis of PTSD requires exposure to a traumatic event (the person has been exposed or witnessed or confronted with one or more events that have involved death or threat of death, or serious injuries or a threat to their physical integrity or that of others), and the fact of having reacted to them with a feeling of intense fear, helplessness or horror.

These events can have different consequences in a person's life, such as leading to the development of posttraumatic stress disorder (PTSD), characterized by symptoms such as re-experiencing the traumatic event, avoidance, negative cognitions and neurovegetative hyperactivation [3]. Although most people exposed to a traumatic event do not develop any mental health difficulties, some individuals exhibit vulnerability factors that have both short and long term effects [4]. As a whole population can experience difficulties following a traumatic event, some groups of people seem to be more at risk than others [5]. Thus, adolescents constitute a population particularly at risk of developing a mental health disorder following a traumatic experience, given that the coping strategies to cope with psychologically trying situations have not yet been conclusively consolidated [5,6].

Studies have shown the peculiarities of the symptoms of PTSD in children and adolescents [7]. One may be surprised by the fact that, having gone through situations of extreme danger, such a person develops PTSD, and another remains psychically unscathed [8]. PTSD is very common in the presence of comorbid psychopathological disorders and among these most common disorders we can find anxiety and depression. Namely, very few studies have been conducted to assess the prevalence of PTSD in Morocco and no study has been done to assess the prevalence of PTSD among school-going adolescents. In our study, we realized the prevalence of PTSD and the prevalence of disorders associated with PTSD (anxiety and depression) among adolescents attending middle schools and high schools in the prefecture of Sale in Morocco and study the risk factors for developing PTSD. We also studied the extent of the impact of PTSD on the well-being of these adolescents.

## Methods

**Study design and setting:** at the level of the prefecture of Salé where the survey was carried out, we randomly selected 10 high schools and 15 middle schools. The study was in the form of a cross-sectional survey from March 2017 until June 2017. With the collaboration of the directors of schools, we had distributed a newsletter to each student explaining the purpose of the study.

**Figure 1:**
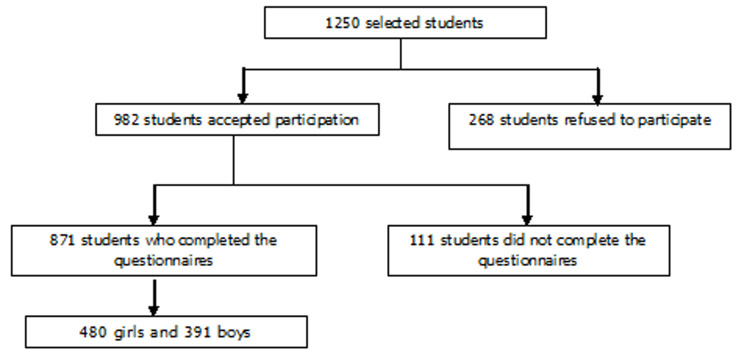
diagram of participant sampling

**Study population:** the study participants were 982 school-age adolescents aged 12 to 17 (Figure 1). The student's acceptance criteria for the survey were to be currently educated, to be present at school during the study, not to exceed the age of 17 years and not to have a mental handicap. Figure 1 describes the recruitment and flow of the participants.

**Data collection:** five questionnaires self-administered which were used successively and all questionnaires were anonymous: i) The socio-demographic data which contained information on the socio demographic profiles of participants. ii) The list of life events: the questionnaire was presents stressful life events with 17 questions according to the DSM -IV [9]. iii) The Children's Posttraumatic Stress Reaction Index (CPTS-RI): The questionnaire was distributed to assess the symptoms of PTSD after exposure to a traumatic event [10]; this is a scale of 20 items intended for children aged 6 to 16. A score between 12 and 24 indicates a low level of PTSD, between 25 and 39 a moderate level, between 40 and 59 a severe level and a score over 60 a very severe level, those with a score below 12 did not present PTSD. iv) The State Trait Inventory Anxiety Form Y (STAIY): the questionnaire was carried out to assess the intensity of adolescent anxiety [11], this scale includes 20 items. A score above 65 indicates very high anxiety, 56 to 65 indicates high anxiety, 46 to 55 indicates medium anxiety, 36 to 45 indicates low anxiety, less than 35 indicates very low anxiety. v) The Children Depression Inventory (CDI): the questionnaire was carried out to assess the intensity of depressive symptoms in adolescents aged 7 to 17 [10], this scale includes 27 items. A score less than 15 proves the absence of depression and a score equal or greater than 15 proves the presence of depression.

**Statistical analysis:** the processing and analysis of all statistical data received by the questionnaires was carried out by the statistical software SPSS version 20. Based on descriptive statistical analysis, the data were presented by number of persons (n) and percentage of persons (%). Continuous variables were expressed as mean and standard deviation. Univariable and multivariable binary logistic regression analyzes were performed to investigate significant factors associated with PTSD. Independent variables that were chosen from univariable analysis to multivariable analysis should have a p-value less than 0.1. The confidence interval (C.I) was set at 95%. Statistical significance was considered at a p-value less than 0.05.

**Ethical considerations:** we had the written approval of the Ministry of National Education and Scientific Research. Informed consents signed by parents or guardians were obtained by the 982 adolescents. The participants were de-identified before statistical analyses.

## Results

**Socio-demographic data:**Table 1 shows the distribution of socio-demographic data and traumatic events. The average age of the students was 14.98 with a standard deviation of 1.497. The average salary of both parents was 2.76 with a standard deviation 1.506, implying that the parents' monthly income was minus 4000 dh (Moroccan dirham) and resulting in low income. The mean frequency of smoking per week was 1.27 with a standard deviation 2.495, the mean frequency of drinking alcohol per week was 0.03 with a standard deviation 0.212, and the average frequency of illicit drug use per week was 0.47 with a standard deviation 1.517. Of the 17 traumatic events listed, all of the students who reported that they did not have the traumatic event of participating in a fight or a war. While the most traumatic event experienced by the students was the sudden and unexpected death of a loved one with 241 (27.7%) students (Table 1).

**Table 1:** the distribution of socio-demographic data and traumatic events

Variable	Number (%)
**Age (years)**	
12-14	334 (38.3)
15-17	537 (61.7)
**Gender**	
Male	391(44.9)
Female	480 (55.1)
**Marital status**	
Married	726 (83.4)
Single	145 (16.6)
**Unemployed father**	25 (2.9)
**Mother without a job**	729 (83.7)
**Parents' salary**	
Under 4000dh	443 (50.9)
Between 4000 and 8000dh	290 (33.3)
More than8000dh	138 (15.8)
**Tobacco consumption**	231 (26.5)
**Alcohol consumption**	25 (2.9)
**Use of illicit drugs**	97 (11.1)
**Traumatic events**	
The sudden and unexpected death of a loved one	241 (27.7)
Another very stressful experience	152 (17.5)
Accident of the public way	82 (9.4)
**Date of event**	
Between 1 month and 6 months	279 (32)
Between 6 months and 1 year	136 (15.6)
Between 1 year and 3 years	196 (22.5)
More than 3 years	260 (29.8)

**Figure 2:**
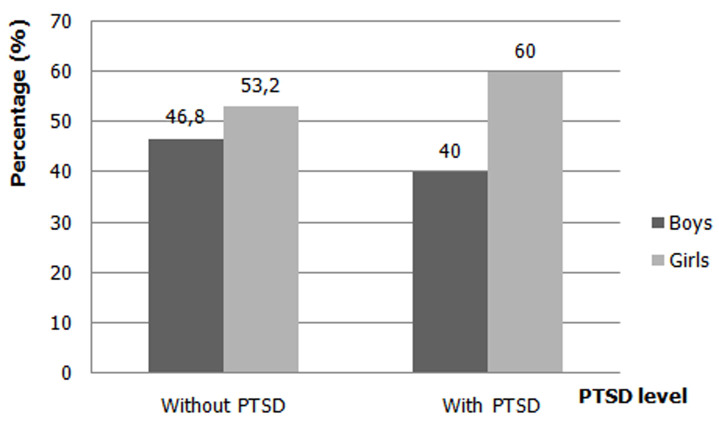
percentage of posttraumatic stress disorder by gender

**Prevalence of posttraumatic stress disorder (PTSD):** using the results of the CPTS-RI scores obtained and among 871 adolescents, we had found 703 (80.7%) students who did not present with posttraumatic stress disorder, 168 (19.3%) students who presented PTSD. Figure 2 shows the percentage of posttraumatic stress disorder by gender. The adolescents who had PTSD, we had found 40% in boys (n = 67), while in girls 60% (n = 101), so girls had a higher percentage of PTSD than boys (Figure 2).

**Prevalence of comorbid disorders:** using the results of the STAIY scores obtained for students with PTSD, we had found 35 (20.83%) students who did not have anxiety, 48 students (28.57%) with low severity level of anxiety, 31 (18.45%) students with moderate severity of anxiety, 33 (19.64%) students with severe severity of anxiety, 21 (12.51%) students with a very severe level of severity of anxiety. So, the students who presented anxiety were 133 (79.17%) students. The adolescents who presented with anxiety, we had found 30.36% (n = 51) in boys, while in girls it was 48.81% (n = 82), so girls have a higher percentage of anxiety than boys. Also using the results of CDI scores obtained for students with PTSD, we found 82 (48.81%) students who did not have depression and 86 (51.19%) students who did have depression. The adolescents who presented with depression, we found 16.1% (n = 27) in boys, while in girls it was 35.1% (n = 59), so the girls had a higher percentage of depression than boys.

**Factors associated with post-traumatic stress disorders:**Table 2 shows univariable and multivariable factors associated with Posttraumatic Stress Disorder (PTSD). The factors independently associated with posttraumatic stress disorder included being a girl which was about 2.1 folds increased odd of PTSD than in boys (AOR=2.113, 95% C.I=1.015-4.399, p=0.046). Besides, being a student at a middle school has about 5.7 odd increase of PTSD compared with their counterparts in the high school (AOR=5.765, 95% C.I=2.262-14.692, p<0.0001). Students whose having a sleep interrupted (AOR=0.142, 95% C.I=0.027-0.745, p=0.021) and students whose having a guilt (AOR=27.378, 95% C.I=6.835-109.663, p<0.0001) were more likely to have PTSD. Also, students whose having a difficulties of memory (AOR=0.157, 95% C.I=0.071-0.346, p<0.0001) and having a difficulties of concentration (AOR=0.041, 95% C.I=0.004-0.392, p=0.006) were more likely to have posttraumatic stress disorder (Table 2). Among the negative effects of the presence of PTSD on the well-being of adolescents, we had found 73% of the students who had declining academic results. The students who repeated the school year were 35.4%. The adolescents who wanted to commit suicide were 5.8%. We had found 27% of the adolescents who use tobacco.

**Table 2:** univariable and multivariable factors associated with posttraumatic stress disorder (PTSD)

Variable	Univariable analysis		Multivariable analysis	
OR (95% CI)	p-value	AOR (95% CI)	p-value
**Age (years)**				
12-14 (ref)				
15-17	1.383 (0.730-2.621)	0.320		
**Gender**				
Male (ref)				
Female	1.689 (1.278-2.232)	<0.0001	2.113 (1.015-4.399)	0.046
**Marital status**				
Married (ref)				
Single	0.981 (0.529-1.818)	0.950		
**Parent's salary (dh)**				
Above 4000 (ref)				
Below 4000	1.892 (1.012-3.536)	0.046	2.082 (0.493-8.793)	0.318
**School level**				
High school (ref)				
Middle school	1.997 (1.506-2.649)	<0.0001	5.765 (2.262-14.692)	<0.0001
**Consumption of tobacco**				
No (ref)				
Yes	1.060 (0.774-1.452)	0.716		
**Consumption of alcohol**				
No (ref)				
Yes	1.465 (0.605-3.546)	0.398		
**Consumption of illicit drug**				
No (ref)				
Yes	1.227 (0.782-1.927)	0.374		
**Sleep interrupted**				
No (ref)				
Yes	0.153 (0.035-0.665)	0.012	0.142 (0.027-0.745)	0.021
**Guilt**				
No (ref)				
Yes	31.832 (11.446-88.529)	<0.0001	27.378 (6.835-109.663)	<0.0001
**Difficulties of memory**				
No (ref)				
Yes	0.141 (0.101-0.197)	<0.0001	0.157 (0.071-0.346)	<0.0001
**Difficulties of concentration**				
No (ref)				
Yes	0.015 (0.002-0.106)	<0.0001	0.041 (0.004-0.392)	0.006

## Discussion

We had found in our study a prevalence of 19.3% in school-going adolescents who had PTSD and 80.7% of school-going adolescents who did not have PTSD. According to the results of our study, the percentage of PTSD among adolescent girls in school was higher compared to those in school, at 60% among girls compared to 40% among boys. Also, we had found in our study a high rate of anxiety level with 79.1% and depression with 51.1% for adolescents with PTSD.

These adolescents were more likely to develop PTSD for several possible causes such as belonging to developing countries with low income such as the country where our survey was conducted [12]. Compared to adults and depending on several criteria such as the type of trauma experienced and the type of study carried out, the percentage of adolescents developing PTSD was high from 25 to 90% [13-15]. Depending on the instruments used, the differences between the populations studied, the type of trauma, the severity and chronicity of symptoms, the prevalence of PTSD had variable [16,17]. Indeed, several studies have shown that girls developed more PTSD than boys [18,19]. PTSD was very frequently encountered in the presence of comorbid psychopathological disorders such as anxiety and depression [20]. Compared to adults, young people were more vulnerable to anxiety and depression [21]. Therefore, girls with PTSD presented more comorbid disorders than boys and indeed, according to several studies, girls had tend to have associated anxiety or depressive disorders more than boys [4,22]. Namely, in our study, for anxiety, in girls it was 48.8% and 30.3% in boys and for depression it was 35.1% in girls against 16.1% in boys.

The sudden and unexpected death of a loved one of adolescents presented the most stressful traumatic event at 27.7% in our study. The traumatic events most experienced by adolescents were the death of a family member, threat of violence, physical bullying at school [6]. For our study, it was found that adolescents experienced more traumatic events with 58.90% than adolescent girls with 41.10%. Regarding our study, the percentage of students who were at least exposed to one traumatic event during their lifetime was 88.69%. Several studies show that boys have more traumatic events than girls [23,24]. The lifetime prevalence was around 100% for adolescents who would have been exposed to at least one traumatic event [6,25,26].

Among adolescents with PTSD, in our study, we had found 17.4% who had difficulty remembering things the adolescent learned in school or at home. We had also found 73% of the students who had declining academic results, 34.5% showed difficulty concentrating and 5.8% of the adolescents who wanted to commit suicide and 39% of young people were prove guilty. In adolescents who suffered from PTSD, it has been observed that there is a presence of shame and guilt for what happened during a traumatic event [27], difficulties concentrating, memory loss, behavioral problems, disturbed sleep and irritability [28]. Reviviscence and difficulty concentrating were believed to be the most frequent symptoms of PTSD in adolescents [29]. The results of some studies showed that adolescents have more memory difficulties [30]. In addition, some people would develop depressive and anxious symptoms as well as risk-taking behaviors, such as problematic substance use [31-33]. Regarding the consumption of tobacco, alcohol and illicit drugs, we had found 27% of the consumption of tobacco and boys consumed significantly more than girls for all psychoactive substances. In adolescents, we had notice the substance abuse, antisocial behavior, social withdrawal, somatic complaints, reduced academic performance, sleep problems, suicidal thoughts and difficulties with concentration [14,25].

Symptoms of PTSD were important influences on daily living of adolescents and these symptoms could persisted for many years and usually get worse when PTSD is left untreated. The results should be taken into consideration by the local authorities. It had preferable to consider a program of support at school level by psychologists and this program were not implemented so far. Because of the prevalence of PTSD found, reliable implications were needed to cure PTSD, the most commonly used PTSD treatments in adolescents are CBT (cognitive behavioral therapy, EMDR (eye movement, desensitization and information reprocessing) and also pharmacotherapy, psycho-education and also the intervention of public and private organizations to support these adolescents to avoid school failure which is a big national problem.

Given the interest of the study of PTSD in school-going adolescents and especially the few studies concerning these adolescents compared to adults. Also, very few studies in Morocco concerning PTSD and no studies for students in high schools and middle schools, that is why we had chosen this type of population to measure the prevalence of PTSD and comorbid PTSD disorders, which could be a great addition to the literature at this level. The knowledge of the impact of had PTSD on students attending school level. The results of this study could be invested by the government to set up a project to support adolescents with psychological difficulties.

The sample could be larger, but there was no financial support for the study. The participation rate could be higher, but parents were not aware of their children's mental problems to encourage them to participate in the study. The STAIY and CDI scales of measures were in French and not in Arabic language, which took me longer to explain the items on each scale to them. The irresponsibility of some school directors who did not respect the work procedure and who had complicated my intervention tasks, for example some students were not present for the first meeting because they were not informed by their directors and I had to replace them which caused a waste of time and even more effort by the investigator. Another limitation is the measurement of PTSD using a self-administered method rather than assessment by a clinician with a well-established structured clinical interview.

## Conclusion

Posttraumatic stress disorder and its comorbid disorders (anxiety and depression) had been found to be present in adolescents in school settings, which hinders the well-being of these adolescents such as poor school performance and the thought of suicide. There were a variety of factors associated with PTSD such as difficulty sleeping, guilt, difficulties of memory and difficulties of concentration, and protective factors were presented for the prevention and early detection of PTSD in these adolescents. To reduce the prevalence of this disorder, effective intervention by local authorities was needed, parents, psychologists and non-governmental organizations to improve the mental health of these adolescents.

### What is known about this topic

- High prevalence of posttraumatic stress disorder in developing countries;
- Very few studies for the prevalence of PTSD in Morocco and the risk factors of PTSD are poorly studied in Morocco;
- Lack of care for school-going adolescents suffering from PTSD.

### What this study adds

- The prevalence of PTSD among adolescents attending schools was 19.3%;
- The prevalence of comorbid disorders of PTSD (anxiety and depression) in school-going adolescents was 79.18% for the anxiety and 51.10% for the depression;
- There were a variety of factors associated with PTSD such as difficulty sleeping, guilt, difficulties of memory and difficulties of concentration.
